# Clinical outcome of bonded partial indirect posterior restorations on vital and non-vital teeth: a systematic review and meta-analysis

**DOI:** 10.1007/s00784-021-04187-x

**Published:** 2021-10-10

**Authors:** Mario Dioguardi, Mario Alovisi, Giuseppe Troiano, Carlo Vito Alberto Caponio, Andrea Baldi, Giovanni Tommaso Rocca, Allegra Comba, Lorenzo Lo Muzio, Nicola Scotti

**Affiliations:** 1 Department of Clinical and Experimental Medicine, University of Foggia, Via Rovelli 50, 71122 Foggia, Italy; 2 Department of Surgical Sciences, Dental School, University of Turin, Via Nizza 230, 10100 Turin, Italy; 3 Division of Cariology and Endodontology, University Clinics of Dental Medicine, University of Geneva, Geneva, Switzerland

**Keywords:** Dental restoration failure, Inlay, Onlay, Overlay, Survival rates, Endodontically treated teeth

## Abstract

**Objectives:**

The survival rate of indirect partial adhesive restorations on vital versus endodontically treated teeth is still controversial. The hypothesis is that there may be a difference in the survival rate of partial adhesive restorations performed on non-vital teeth compared to vital teeth.

**Materials and methods:**

This systematic review was conducted following the PRISMA guidelines. The considered clinical studies investigated the outcomes of adhesive inlays, onlays, and overlays conducted over the past 40 years, focusing on Kaplan–Meier survival curves to calculate the hazard ratio (primary objective) and the survival rate (secondary objective) between vital and non-vital teeth. The risk of bias was assessed using the Newcastle–Ottawa Scale. Studies included in the review were identified through bibliographic research on electronic databases (“PubMed,” “Scopus,” “Cochrane Central Register of Controlled Trial,” and “Embase”). The *K* agreement between the two screening reviewers was evaluated.

**Results:**

A total of 55,793 records were identified on PubMed, Scopus, and other bibliographic sources, and after the application of the eligibility and inclusion criteria, eight articles were included for qualitative analysis and six for quantitative analysis. The meta-analysis of the primary and secondary outcomes demonstrated that hazard ratios (HR = 8.41, 95% CI: [4.50, 15.72]) and survival rates (OR = 3.24, 95% CI: [1.76, 5.82]) seemed more favorable for indirect partial adhesive restorations on vital teeth than for those on endodontically treated teeth.

**Conclusions:**

Within the limits of this study, these findings suggest that the risk of failure of indirect partial adhesive restorations on endodontically treated teeth is higher than on vital teeth.

**Clinical relevance:**

The use of partial adhesive restorations on vital and endodontically treated teeth showed different long-term clinical outcomes.

## Introduction

The clinical failure of adhesive restorations still raises strong interest in the scientific literature, especially when endodontically treated teeth (ETT) are considered. In particular, it is important to identify the most frequent causes of failure to better prevent their long-term occurrence. The main sources of direct or indirect restoration failure in vital or endodontically treated teeth are found in biological and mechanical causes, such as secondary caries, hypersensitivity, pulp pathology, chronic and acute apical periodontitis, tooth and root fractures, ceramic or resin composite chipping, and loss of retention or adhesion [1]. However, failures may have completely different clinical implications: they can lead to a re-intervention that aims at tooth healing and consequent maintenance or to tooth loss.

In vital posterior teeth, the annual restorative failure rate could vary from 1 to 3% in medium- and large-size cavities, while the failure rate may range from 2 to 12.4% in endodontically treated teeth [2–4]. da Rosa Rodolpho et al. concluded that, during a 17-year monitoring period, 5.1% of restored teeth needed replacement due to endodontic reasons, which could negatively contribute to the reduction of the tooth survival rate [5]. Indeed, recent studies have reported that posterior tooth longevity mainly depends on the amount of remaining tooth structure and the variation of the physical–mechanical properties of the dentin over years [6–8]. Thus, the efficacy of restorative procedures in preserving sound teeth and minimizing root tissue loss is crucial [9].

In the past, there was the opinion that ETT needed a root canal post and full coverage crown rehabilitation [10, 11]. Aquilino and Caplan showed that cuspal coverage could increase up to six times the survival rate of non-vital posterior teeth [12]. Therefore, the full crown has been considered the gold standard therapeutic approach for large cavities in ETT for years [13]. However, full crown preparations tend to remove a large amount of healthy dental tissue from teeth that have already lost a huge quantity of sound tooth structure due to pathology and endodontic procedures [14]. Hence, the majority of recent studies have focused more on partial direct or indirect bonded restorations, which ensure higher sound tissue preservation than traditional fixed full crowns [15, 16]. As a consequence of this paradigm shift, direct and indirect partial bonded restorations, such as inlays, onlays, overlays, and endocrowns, have been proposed for the rehabilitation of ETT as valid therapeutic alternatives to conventional prosthetic solutions [17–20].

Regarding bonded partial indirect restorations on vital and non-vital teeth, the data emerging from the literature are partially merged and sometimes in contrast. A previous study reported that the 11-year success rate of inlays and onlays is 80% [21], while Skupien et al. showed that among a population of 69 inlays, only two failed, leading to extraction of the dental element, with a whole success rate of 85.5% at 9 years [22]. However, Reiss et al. after a follow-up of 16.7 years reported 28 failures out of 77 ETT with a survival rate of 63% [23]. Furthermore, Vagropoulou et al. investigated the survival of inlays and onlays versus complete coverage restorations, finding an overall 5-year rate of 90% [24]. Systematic reviews have repeatedly investigated the survival of inlays, onlays, and overlays. Morimoto et al. reported a survival rate of 92–95% for glass-reinforced ceramics and feldspathic porcelains at 5 years and 91% at 10 years, without distinction between vital and non-vital teeth, identifying the main failure cause as fracture/chipping of the restoration material [25]. Recently, Sampaio et al. reported the survival rate of the CAD/CAM inlays as 97% after 5 years and 89% after 10 years. In particular, the rate was 95% after 5 years for pressable ceramics, and for stratified ceramics, it was 88% after 5 years and 93% after 10 years [26]. However, previous systematic reviews, with or without meta-analyses, did not investigate the survival rate of partial indirect bonded posterior restorations on vital teeth compared with ETT. Thus, the present study null hypothesis is that there is no difference in the survival rate of partial indirect adhesive restorations performed on non-vital teeth compared to vital teeth.

## Materials and methods

The following systematic review was conducted based on the indications of the Preferred Reporting Items for Systematic Reviews and Meta-Analyses (PRISMA) statement [27] and was registered in PROSPERO: CRD42020204095.

The study was constructed on the population, intervention, control, and outcome (PICO) questions: patient (patients who need restorative treatment with inlay, onlay, and overlay on ETT), intervention (inlay, onlay, and overlay), control (patients with inlay, onlay, and overlay on vital teeth), and outcome (hazard ratio and survival rate for inlay, onlay, and overlay between vital and non-vital teeth); a scientific question was asked: What is the hazard ratio of failure of indirect partial restorations in ETT compared to those placed on vital teeth?

### Eligibility criteria

The considered clinical studies investigated the survival rate of adhesive inlays, onlays, and overlays published in English and conducted over the past 40 years. We decided to focus on the last 40 years due to partial restoration manufacturing techniques that have undergone a profound change from gold casts to more recent CAD-CAM techniques. Investigating clinical studies with follow-ups published before 1980 would have led to an increase in heterogeneity, with a high risk of bias. Bibliographies of previously published systematic reviews on similar topics were checked to find articles for potential inclusion in this study.

After an initial screening of abstracts identified on the evaluated databases, the potentially eligible articles were qualitatively evaluated to investigate the survival rate of inlays, onlays, and overlays on both vital and ETT, focusing on the research of the studies that reported hazard ratios or Kaplan–Meier survival curves to allow the calculation of the hazard ratio of vital to non-vital teeth. The potentially eligible articles were eventually subjected to a full-text analysis to verify their eligibility for inclusion in both qualitative and quantitative analyses.

The inclusion and exclusion criteria applied in the full-text analysis were as follows:

- Includes all articles that report data on the inlays, onlays, and overlays hazard ratio between vital and non-vital teeth or the Kaplan–Meier survival curves (primary outcome);
- Includes all studies that report data on survival and success rates of inlays, onlays, and overlays on non-vital teeth (secondary outcome);
- Excludes all studies and articles that do not report data on the survival of partial adhesive restorations on ETT; studies reporting data on the same sample already investigated in previous studies; are published in a language other than English; are published prior to 1979; with a high risk of bias.

### Research and screening methodology

Studies included in the review were identified through bibliographic research on electronic databases. The literature search was conducted on the search engines “PubMed,” “Scopus,” “Cochrane Central Register of Controlled Trial,” and “Embase.” The database search was conducted between June 1, 2020, and June 9, 2020, and the last search for a partial update of the literature was conducted on July 18, 2021. The details regarding the search terms and combination strategies used in the literature review are reported in Table 1.

**Table 1:** Complete overview of the search methodology. Articles selected for qualitative analysis: 8. Articles selected for quantitative analysis: 6

Database	Keywords	Search details	Number of records	Number of records after restriction by year of publication (last 40 years)	Articles after removing overlaps articles	Number of remaining articles related to the topic of inlay or onlay	Number of articles remaining after applying the inclusion and exclusion criteria	Number of articles included for the first outcome	Number of articles included for the second outcome
PubMed	inlay AND onlay AND overlay	((("inlays"[MeSH Terms] OR "inlays"[All Fields]) OR "inlay"[All Fields]) AND (((("inlays"[MeSH Terms] OR "inlays"[All Fields]) OR "onlay"[All Fields]) OR "onlays"[All Fields]) OR "onlayed"[All Fields])) AND ((("overlay"[All Fields] OR "overlayed"[All Fields]) OR "overlaying"[All Fields]) OR "overlays"[All Fields])	75	69					
PubMed	inlay OR onlay OR overlay AND Vital	("inlays"[MeSH Terms] OR "inlays"[All Fields] OR "inlay"[All Fields] OR ("inlays"[MeSH Terms] OR "inlays"[All Fields] OR "onlay"[All Fields] OR "onlays"[All Fields] OR "onlayed"[All Fields]) OR ("overlay"[All Fields] OR "overlayed"[All Fields] OR "overlaying"[All Fields] OR "overlays"[All Fields])) AND ("vital signs"[MeSH Terms] OR ("vital"[All Fields] AND "signs"[All Fields]) OR "vital signs"[All Fields] OR "vital"[All Fields] OR "vitally"[All Fields] OR "vitals"[All Fields])	180	163					
PubMed	inlay OR onlay OR overlay AND Dental	("inlays"[MeSH Terms] OR "inlays"[All Fields] OR "inlay"[All Fields] OR ("inlays"[MeSH Terms] OR "inlays"[All Fields] OR "onlay"[All Fields] OR "onlays"[All Fields] OR "onlayed"[All Fields]) OR ("overlay"[All Fields] OR "overlayed"[All Fields] OR "overlaying"[All Fields] OR "overlays"[All Fields])) AND ("dental health services"[MeSH Terms] OR ("dental"[All Fields] AND "health"[All Fields] AND "services"[All Fields]) OR "dental health services"[All Fields] OR "dental"[All Fields] OR "dentally"[All Fields] OR "dentals"[All Fields])	7894	6878					
PubMed	inlay OR onlay OR overlay AND tooth	("inlays"[MeSH Terms] OR "inlays"[All Fields] OR "inlay"[All Fields] OR ("inlays"[MeSH Terms] OR "inlays"[All Fields] OR "onlay"[All Fields] OR "onlays"[All Fields] OR "onlayed"[All Fields]) OR ("overlay"[All Fields] OR "overlayed"[All Fields] OR "overlaying"[All Fields] OR "overlays"[All Fields])) AND ("teeth s"[All Fields] OR "teeths"[All Fields] OR "tooth"[MeSH Terms] OR "tooth"[All Fields] OR "teeth"[All Fields] OR "tooth s"[All Fields] OR "tooths"[All Fields]))	3197	2866					
PubMed	partial Crown OR endo crown AND Vital	(((("partial"[All Fields] OR "partials"[All Fields]) AND ("crown s"[All Fields] OR "crowned"[All Fields] OR "crowns"[MeSH Terms] OR "crowns"[All Fields] OR "crown"[All Fields])) OR ("endo"[All Fields] AND ("crown s"[All Fields] OR "crowned"[All Fields] OR "crowns"[MeSH Terms] OR "crowns"[All Fields] OR "crown"[All Fields]))) AND ("vital signs"[MeSH Terms] OR ("vital"[All Fields] AND "signs"[All Fields]) OR "vital signs"[All Fields] OR "vital"[All Fields] OR "vitally"[All Fields] OR "vitals"[All Fields]))	121	108					
PubMed	partial Crown OR endo crown AND Dental	(((("partial"[All Fields] OR "partials"[All Fields]) AND ("crown s"[All Fields] OR "crowned"[All Fields] OR "crowns"[MeSH Terms] OR "crowns"[All Fields] OR "crown"[All Fields])) OR ("endo"[All Fields] AND ("crown s"[All Fields] OR "crowned"[All Fields] OR "crowns"[MeSH Terms] OR "crowns"[All Fields] OR "crown"[All Fields]))) AND ("dental health services"[MeSH Terms] OR ("dental"[All Fields] AND "health"[All Fields] AND "services"[All Fields]) OR "dental health services"[All Fields] OR "dental"[All Fields] OR "dentally"[All Fields] OR "dentals"[All Fields]))	4704	3799					
PubMed	partial Crown OR endo crown AND tooth	((("partial"[All Fields] OR "partials"[All Fields]) AND ("crown s"[All Fields] OR "crowned"[All Fields] OR "crowns"[MeSH Terms] OR "crowns"[All Fields] OR "crown"[All Fields])) OR ("endo"[All Fields] AND ("crown s"[All Fields] OR "crowned"[All Fields] OR "crowns"[MeSH Terms] OR "crowns"[All Fields] OR "crown"[All Fields]))) AND ("teeth s"[All Fields] OR "teeths"[All Fields] OR "tooth"[MeSH Terms] OR "tooth"[All Fields] OR "teeth"[All Fields] OR "tooth s"[All Fields] OR "tooths"[All Fields])	2537	2216					
PubMed	inlay OR onlay OR Overlay AND endodontic	(((("inlays"[MeSH Terms] OR "inlays"[All Fields]) OR "inlay"[All Fields]) OR (((("inlays"[MeSH Terms] OR "inlays"[All Fields]) OR "onlay"[All Fields]) OR "onlays"[All Fields]) OR "onlayed"[All Fields])) OR ((("overlay"[All Fields] OR "overlayed"[All Fields]) OR "overlaying"[All Fields]) OR "overlays"[All Fields])) AND ((((("endodontal"[All Fields] OR "endodontic"[All Fields]) OR "endodontical"[All Fields]) OR "endodontically"[All Fields]) OR "endodontics"[MeSH Terms]) OR "endodontics"[All Fields]	428	369					
PubMed	partial Crown OR endo crown AND endodontic	"((("partial"[All Fields] OR "partials"[All Fields]) AND (((("crown s"[All Fields] OR "crowned"[All Fields]) OR "crowns"[MeSH Terms]) OR "crowns"[All Fields]) OR "crown"[All Fields])) OR ("endo"[All Fields] AND (((("crown s"[All Fields] OR "crowned"[All Fields]) OR "crowns"[MeSH Terms]) OR "crowns"[All Fields]) OR "crown"[All Fields]))) AND ((((("endodontal"[All Fields] OR "endodontic"[All Fields]) OR "endodontical"[All Fields]) OR "endodontically"[All Fields]) OR "endodontics"[MeSH Terms]) OR "endodontics"[All Fields]	551	495					
Scopus	inlay OR onlay OR Overlay AND endodontic; partial Crown OR endo crown AND endodontic; inlay AND onlay AND overlay; inlay OR onlay OR overlay AND Vital; inlay OR onlay OR overlay AND Dental; inlay OR onlay OR overlay AND tooth; partial Crown OR endo crown AND Vital; partial Crown OR endo crown AND Dental	Title Abstract Keyword	20,594	17,600					
Other bibliographic sources (literature reviews)	“Systematic reviews”	Sampaio et al. 2019 [26], Morimoto et al. 2016 [25], Vagropoulou et al. 2018 [24], Abduo et al. 2018 [28]	266	264					
Cochrane Central Register of Controlled Trial	inlay OR onlay OR Overlay AND endodontic; partial Crown OR endo crown AND endodontic; inlay AND onlay AND overlay; inlay OR onlay OR overlay AND Vital; inlay OR onlay OR overlay AND Dental; inlay OR onlay OR overlay AND tooth; partial Crown OR endo crown AND Vital; partial Crown OR endo crown AND Dental;	Title Abstract Keyword	3968	3953					
Embase	inlay OR onlay OR overlay AND Vital; inlay OR onlay OR overlay AND Dental; inlay OR onlay OR overlay AND tooth; partial Crown OR endo crown AND vital; partial Crown OR endo crown AND Dental; partial Crown OR endo crown AND tooth inlay OR onlay OR overlay AND endodontic; partial Crown OR endo crown AND endodontic	Title Abstract Keyword	11,278	10,467					
Total records			55,793	49,247	10,770	692	31	5	6

The keywords for the search and their combinations were decided before the identification phase by common agreement between two reviewers (with the task of selecting potentially eligible articles). Overlaps were removed through the use of EndNote 8.0.

The records obtained were subsequently examined by two independent reviewers (M.D. and M.A.) and a third reviewer (G.T.) acted as a decision-maker in situations of doubt.

The screening included an analysis of the title and the abstract to eliminate records not related to the topics of the review. After the screening phase, complete texts of the articles were analyzed, from which the ones eligible for the qualitative analysis and the inclusion in the meta-analysis for the two outcomes were identified. Data sought by the three reviewers in the included studies were as follows:

1. Primary outcome: Hazard ratio between inlays, onlays, and overlays indirect adhesive restorations on non-vital and vital teeth (reviewers sought Kaplan–Meier survival curves for inlays on vital and non-vital teeth).
2. Secondary outcome: Survival rate of inlays, onlays, and overlays indirect adhesive restorations on vital and non-vital teeth (the reviewers searched for all failures regarding inlays, onlays, and overlays on vital and non-vital teeth).

The *K* agreement between the two screening reviewers was 0.84 [29]. The *K* agreement was based on the formulas in the *Cochrane handbook for systematic reviews of interventions* [30].

### Statistical analysis protocol

The protocol used for the meta-analysis was based on the indications of the *Cochrane handbook for systematic reviews of interventions* (Chapter 11, Sect. 11.3.2).

To calculate the hazard ratio for the log hazard ratio and the variance in the included articles that did not report the data, the value was extracted using the Tienery method [31] using the software Engauge Digitizer and the Microsoft Excel spreadsheet created by Matt Sydes for the extraction of summary statistics of literature published for survival endpoints [32].

The extraction of the data and the reporting methods used follow the indications of the *Cochrane handbook for systematic reviews of interventions* Chapter 7 (Selection of Studies and Data Collection), specifically from pages 152 to 182 [30]. The data extraction, performed by two independent reviewers, is summarized and reported in the tables in the “Results” section and subsequently included in the statistical analysis programs.

The software Reviewer Manager 5.4 (Cochrane Collaboration, Copenhagen, Denmark) was used for the meta-analysis, and in particular for pooled hazard ratio, pooled odds ratios (OR), confidence intervals, and inverse of variance.

The Newcastle–Ottawa Scale for case–control studies was used to assess the risk of bias in the included studies in primary and secondary outcomes [33]. The risk of bias assessment for the included articles was conducted by three reviewers, two of whom independently rated the articles, while in case of disagreement, a third reviewer comes to discuss it.

The presence of heterogeneity was assessed by calculating the Higgins index (*I*^2^); if such a measure proved to be higher than 50%, the rate of heterogeneity was considered high. Pooled results of the meta-analysis are represented as forest plots for each of the analyzed outcomes.

We used the GRADE pro-Guideline Development Tool online software (GRADEpro GDT, Evidence Prime, Hamilton, ON) to evaluate the quality of evidence.

## Results

A total of 1621 records were identified on PubMed, Scopus, and other bibliographic sources (reference of systematic reviews concerning the topics of inlays, onlay, and overlays, Table 1). After screening and applying the eligibility and inclusion criteria, the following eight articles were included for qualitative analysis and six for quantitative analysis:

- Five articles for the primary outcome: Beier et al. 2012 [34], van Dijken et al. 2010 [35], Reiss et al. 2006 [23], Bresser et al. 2019 [36], Stoll et al. 2010 [37].
- Six articles for the secondary outcome: Beier et al. 2012 [34], van Dijken et al. 2010 [35], Reiss et al. 2006 [23], Bresser et al. 2019 [36], Stoll et al. 2010 [37], Schulte et al. 2005 [38].
- Two articles were included only for qualitative analysis: Homsy et al. 2015 [39], Studer et al. 2000 [40].

**Fig. 1:**
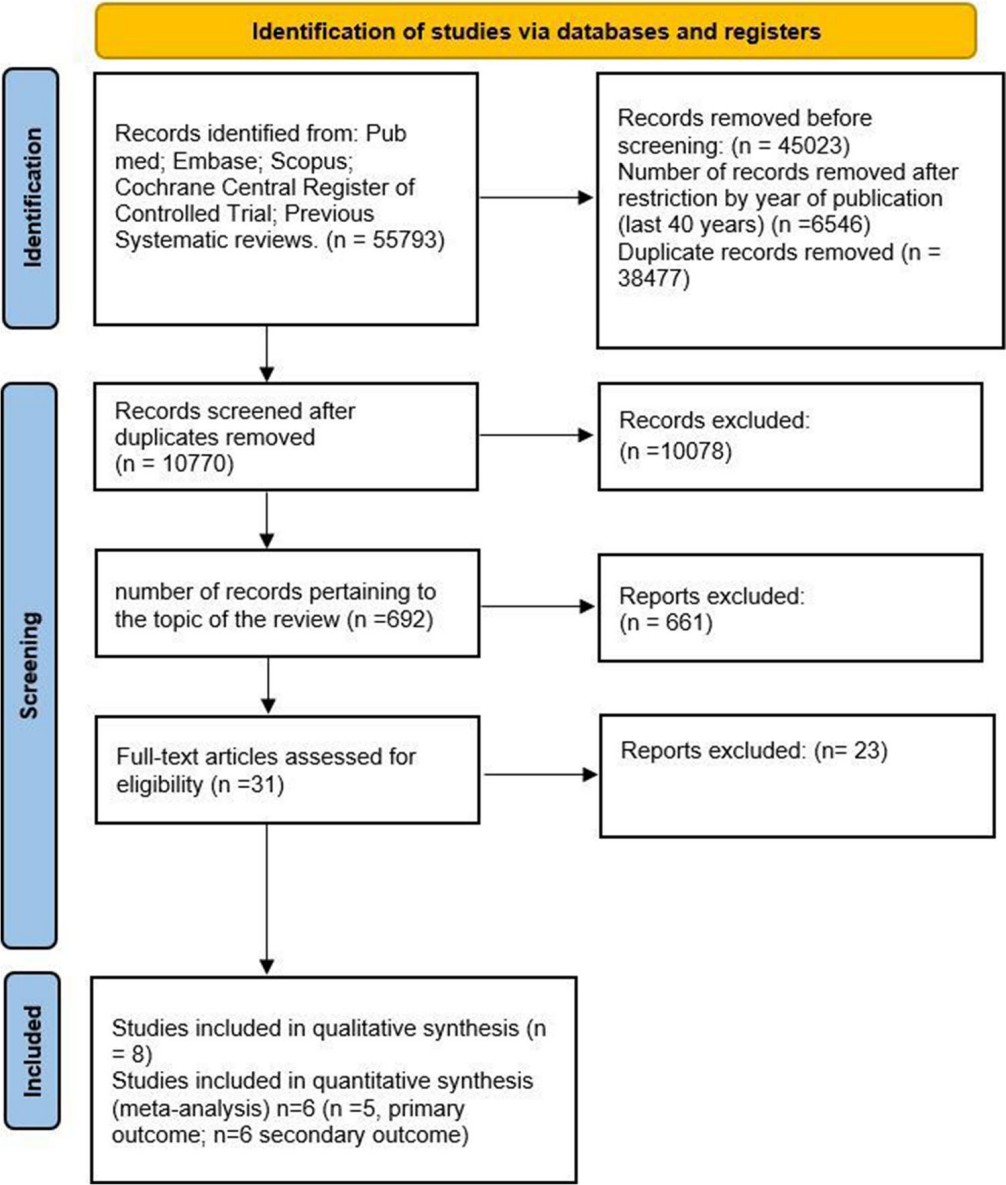
Flowchart of the different phases of the systematic review

The entire selection and screening procedures are described in the PRISMA flowchart (Fig. 1).

### Study characteristics and data extraction

The data that have been extracted from the included studies include the journal (year of publication, first author); the type of clinical study conducted (retrospective, prospective, observational, randomized clinical trial, case–control); the type of rehabilitation performed (inlays, onlays, overlays) and the material (porcelain, glass ceramic, composite, lithium disilicate); the period of inclusion and the average follow-up of the patients; the number of inlays, onlays, and overlays performed on vital and non-vital teeth; and the number of failures. Furthermore, the hazard ratios of vital to ETT were extracted, and images of the survival curves of the indirect restorations were acquired. The data extracted for the two outcomes are reported in Tables 2 and 3.

**Table 2:** Primary and secondary outcomes (the data reported concern the characteristics of the included studies, with the related data on the hazard ratio and the number of failures of indirect restorations)

References	Type of study	Country	Inclusion period	Follow-up	Mean age of patients	Gender	Material	Non-vital teeth		Vital		Hazard ratio	Error standard
								Total	Failure	Total	Failure		
Beier et al., 2012 [34]	Retrospective study	Austria	1987–2009	12 y	46.2 ± 12.5 years; (range: 14–72)	74 females; 46 males	Glass–ceramic	9	5	538	23	20,34	0.531494
van Dijken et al., 2010 [35]	Prospective study	Sweden	1992–1998	15 y	52 years; (range: 26–81)	75 females; 46 males	Dentin-bonded ceramic partial and complete coverages	41	16	187	39	3.85	0.39777
Reiss et al., 2006 [23]	Clinical long-term study	Germany	1987–1990	16.7 y	33 years; (range: 12 –70)	\	Glass–ceramic	77	28	934	94	6	0.2251315
Bresser et al., 2019 [36]	Clinical study	Switzerland	2007–2016	12 y	61.6 years; (range: 30–106)	78 females; 42 males	Indirect ceramic restorations	45	3	152	5	6,33	0.793051
Stoll et al., 2010 [37]	Observational study	Germany	1991–2001	10 y	Male: 38.83 ± 12.12 years; female: 38.08 ± 11.926 years	333 females; 310 males	Glass–ceramic	36	7	1558	46	17,87	0.466252
Schulte et al., 2005 [38]	Retrospective study	Germany	1993–2002	9.6 y	35.4 ± 11.0 years; (range: 17–64)	252 females; 138 males	Glass–ceramic	46	1	764	26	\	\
Homsy et al. 2015 [39]	Clinical study	Lebanon	\	2 y	45 years; (range:18–78)	14 females; 12 males	Glass–ceramic	27	0	14	0	\	\
Studer et al. 2000 [40]	Retrospective study	Switzerland	1950	50 y	56.2 ± 11.4 years; (range: 82.7–38.0)	22 females; 28 males	Partial gold restorations	29	12	274	30	\	\

^1^Excluded from the quantitative analysis because it showed a follow-up duration of only 2 years compared to other included studies that reported an average follow-up period of 12 years

^2^Excluded from the quantitative analysis because it treated partial gold restorations without any adhesive bonding mechanism

**Table 3:** Assessment of risk of bias within the studies (Newcastle–Ottawa Scale) with scores of 7–12 = low quality, 13–20 = intermediate quality, and 21–24 = high quality

		Selection			Comparability		Exposure		Score	Outcome
Reference	Definition of cases	Representativeness of cases	Selection of controls	Definition of controls	Comparability of cases and controls on the basis of the design or analysis	Ascertainment of exposure	Same method of ascertainment for cases and controls	Non-response rate		
Beier et al., 2012 [34]	2	2	3	3	3	3	3	0	19	Primary, secondary
van Dijken et al., 2010 [35]	2	2	3	3	3	3	3	3	22	Primary, secondary
Reiss et al., 2006 [23]	2	2	2	3	3	3	3	0	18	Primary, secondary
Bresser et al., 2019 [36]	2	3	3	3	3	3	3	1	21	Primary, secondary
Stoll et al., 2010 [37]	2	2	3	3	3	3	3	3	22	Primary, secondary
Schulte et al., 2005 [38]	2	2	2	3	3	3	3	0	18	Secondary
Homsy et al. 2015 [39]^**1**^	2	2	2	2	2	1	3	2	16	\
Studer et al. 2000 [40]^**1**^	2	3	2	2	3	3	3	3	21	\

^1^Excluded

### Risk of bias

The risk of bias was assessed through the Newcastle–Ottawa Case–Control Scale, modified by the authors, as demonstrated in previous systematic reviews with meta-analysis [41, 42]. The results are reported in Table 3. For each category, a value of one to three was assigned (one = low and three = high).

Studies presenting a high risk of bias were not included in the meta-analysis. Articles with a high risk of bias were excluded from the scale and eliminated during the inclusion phase. Other articles were excluded due to the investigated outcomes or because they presented the same data and samples. The bias risk assessment for the eight included articles was conducted by M.D., G.T., and M.A.

**Fig. 2:**
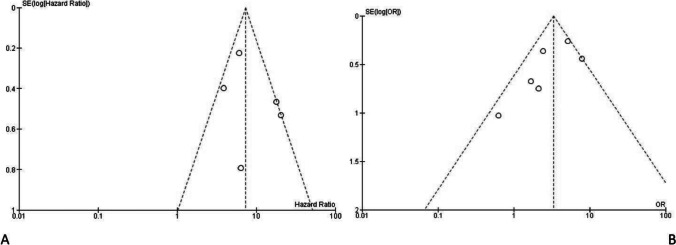
Funnel plots of the evaluation of heterogeneity for the (**A**) primary and (**B**) secondary outcomes

The heterogeneity that emerged from the meta-analysis was average; for the first outcome, we had an *I*^2^ of 68% and for the second outcome, a heterogeneity with an *I*^2^ of 50%. Acceptable heterogeneity values were also confirmed by a funnel plot for the first two outcomes (Fig. 2).

The study by Homsy et al. (2015) [39] was excluded from the quantitative analysis because it showed a follow-up duration of only 2 years compared to other included studies that reported an average follow-up period of 12 years. The study by Studer et al. (2000) [40] was excluded from the quantitative analysis because it treated partial gold restorations without any adhesive bonding mechanism. Therefore, the inclusion would have been a source of bias.

The authors also used GRADE pro-GDT to evaluate the quality of the primary outcomes and secondary outcomes (Table 4). The results suggested that the quality of evidence is moderate for the first outcome and high for the second outcome.

**Table 4:** Evaluation of GRADE pro GDT

GRADE pro GDT											
Certainty assessment							Summary of findings				
Participants (studies) Follow-up	Risk of bias	Inconsistency	Indirectness	Imprecision	Publication bias	Overall certainty of evidence	Study event rates (%)		Relative effect (95% CI)	Anticipated absolute effects	
							With vital	With non-vital		Risk with vital	Risk difference with non-vital
Hazard ratio between inlays, onlays, and overlays indirect adhesive restorations on non-vital and vital teeth											
3577 (5 observational studies)	Serious ^a^	Not serious	Not serious	Serious ^b^	Very strong association all plausible residual confounding would suggest spurious effect, while no effect was observed	⨁⨁⨁◯ MODERATE	414/3369 (12.3%)	59/208 (28.4%)	**HR 8.41** (4.50 to 15.72)	123 per 1.000	**545 more per 1.000** (from 323 to 750 more)
Survival rate of inlays, onlays, and overlays indirect adhesive restorations on vital and non-vital teeth											
4387 (6 observational studies)	Serious ^a^	Not serious	Not serious	Not serious	Very strong association all plausible residual confounding would suggest spurious effect, while no effect was observed	⨁⨁⨁⨁ HIGH	440/4133 (10.6%)	60/254 (23.6%)	**OR 3.20** (1.76 to 5.82)	106 per 1.000	**170 more per 1.000** (from 67 to 303 more)

*CI*, confidence interval; *HR*, hazard ratio; *OR*, odds ratio

^a^High heterogeneity between the studies due to the differences between the materials and the methods adopted for the execution of the restorations

^b^To calculate the hazard ratio for the log hazard ratio and the variance in the included articles that did not report the data, the value was extracted using the Tienery method using the software Engauge Digitizer and the Microsoft Excel spreadsheet created by Matt Sydes for the extraction of summary statistics of literature published for survival endpoints. Although this method is reproducible, it is not free from inaccuracies

### Meta-analysis

**Fig. 3:**
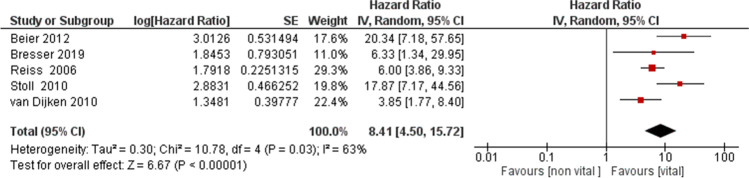
A forest plot of the meta-analysis of the primary outcome

The meta-analysis of the primary outcome (hazard ratio between inlays, onlays, and overlays on vital and ETT) showed heterogeneity with *I*^2^ of 63% (random effects model was applied). The results reported in the forest plot showed that the hazard ratio seemed more favorable for indirect partial adhesive restorations on vital teeth than for those on ETT (HR = 8.41, 95% CI: [4.50, 15.72]) (Fig. 3).

**Fig. 4:**
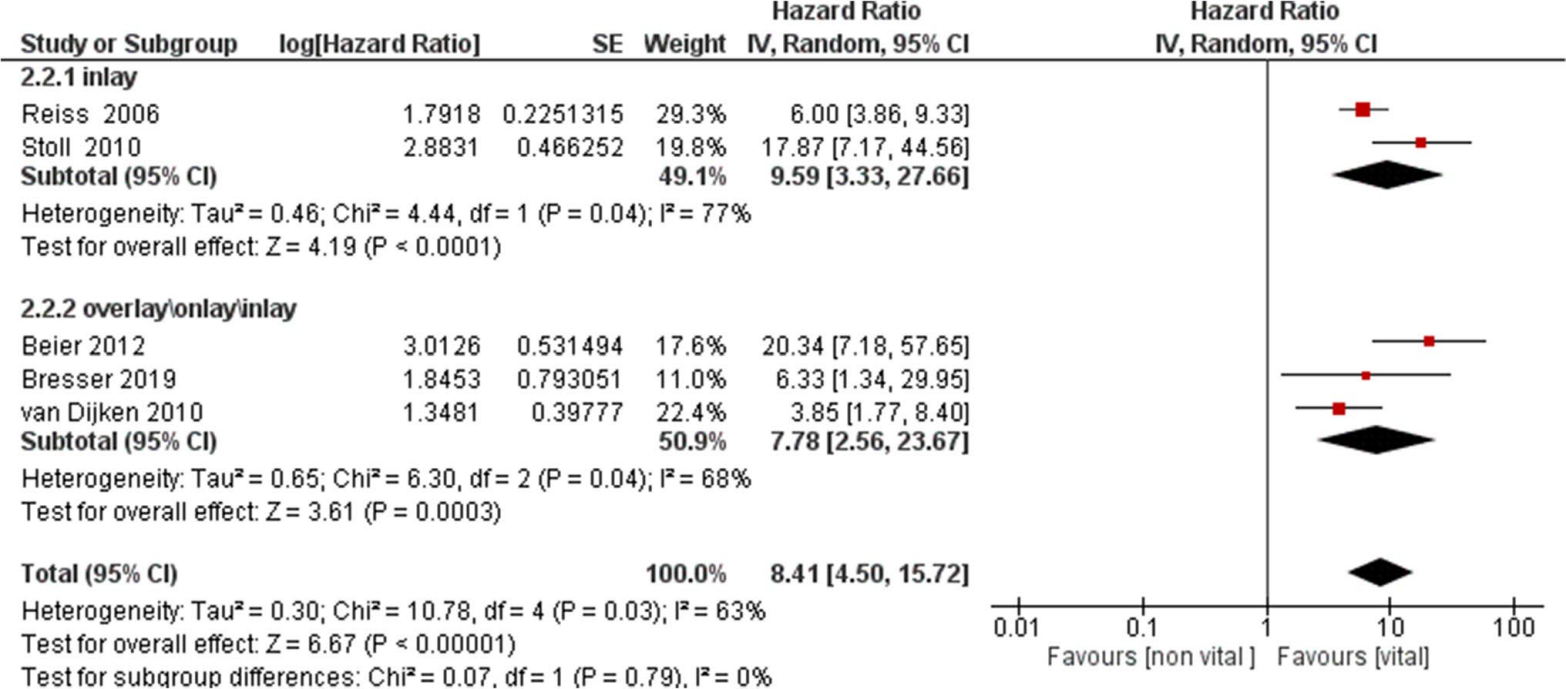
A forest plot of the analysis of the subgroups of the primary outcome

In addition, an analysis of the subgroups was conducted based on the type of restoration. Subgroup: inlay ; Subgroup: overlay\onlay\inlay (Fig. 4). The results reported for the first subgroup (inlay) (HR = 9.59, 95% CI: [3.33, 27.66], for the second subgroup (overlay\onlay\inlay) (HR = 7.78, 95% CI: [2.56, 23.67]), confirmed the same trend.

**Fig. 5:**
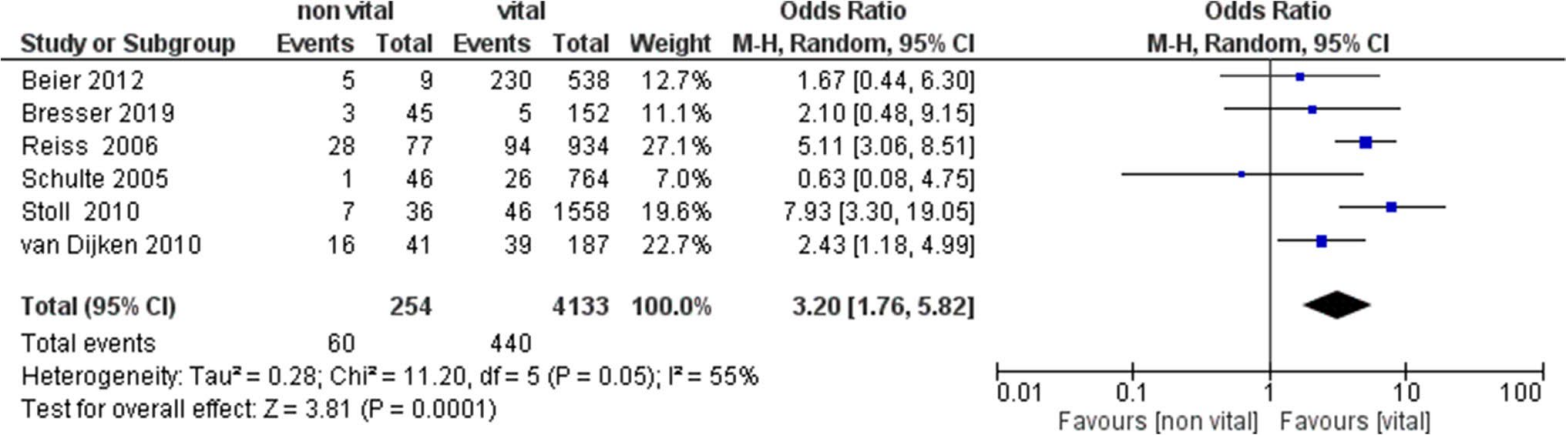
A forest plot of the meta-analysis of the secondary outcome

The meta-analysis of the secondary outcome (survival rate of inlays, onlays, and overlays on non-vital and vital teeth) showed heterogeneity with *I*^2^ of 55% (random effects model was applied). The results reported in the forest plot indicated that the pooled odds ratio seemed more favorable for indirect partial adhesive restorations on vital teeth than for those on ETT (OR = 3.24, 95% CI: [1.76, 5.82]) (Fig. 5).

**Fig. 6:**
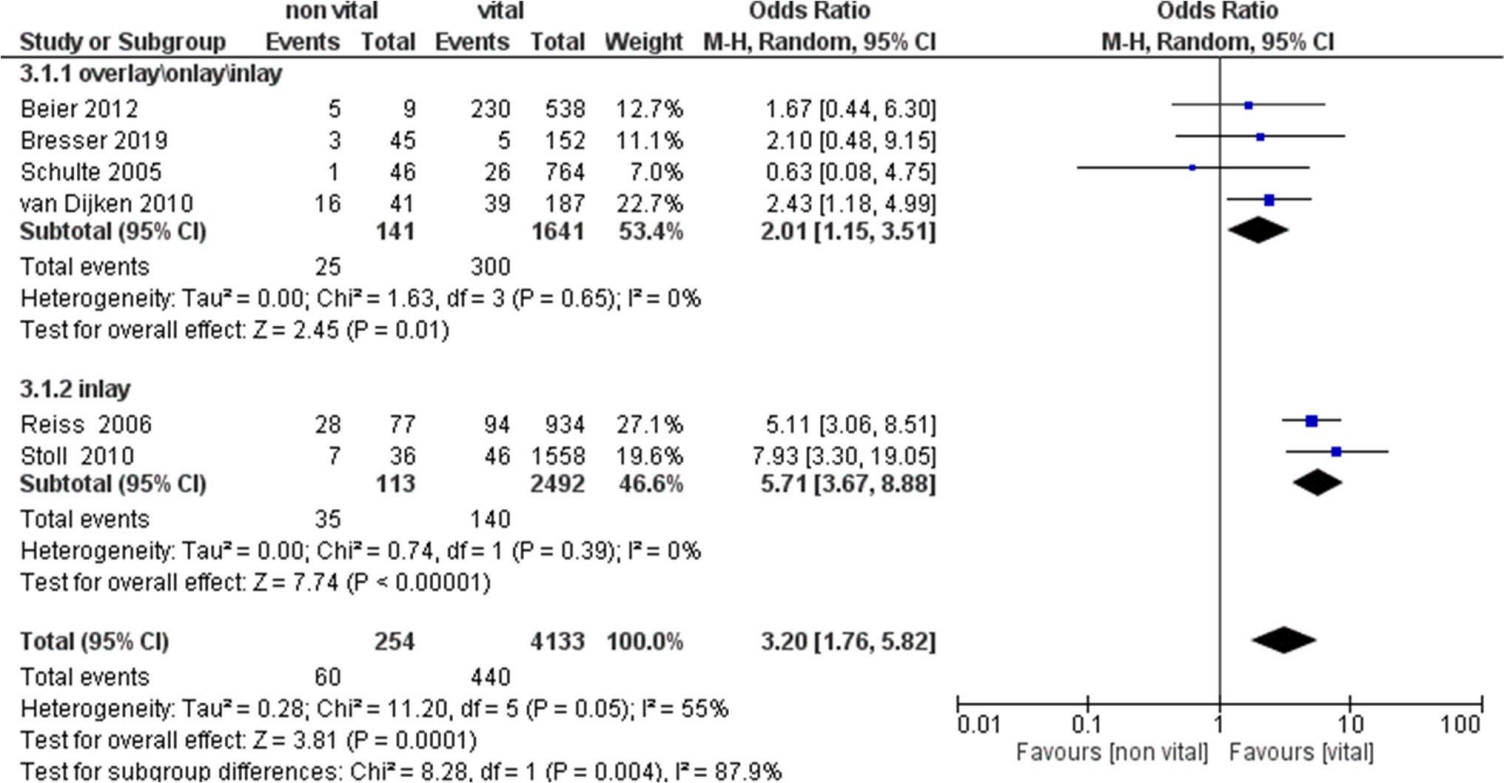
A forest plot of the analysis of the subgroups of the secondary outcome

The analysis of the 2 subgroups reported the following results: Subgroups inlay (OR = 5.71, 95% CI: [3.67, 8.88]); Subgroup overlay\onlay\inlay (OR = 2.01, 95% CI: [1.15, 3.51]) (Fig. 6).

## Discussion

Indirect adhesive solutions represent an increasingly used restorative option and deserve scientific interest that certifies their clinical effectiveness in the rehabilitation of vital and non-vital teeth. It is important to note that an indirect adhesive solution on a vital tooth is necessary in cases in which a dental element is affected by destructive carious lesions or coronal fractures that compromise a large portion of it [5]. In these cases, the tooth triggers defense processes toward the pulp organ, which alters its histological composition, especially at the level of deep dentin [43]. Matrix metalloproteinases (MMPs) can be induced by dentinal demineralization and polymorphonuclear neutrophil inflammatory response to invading microorganisms [44]. However, MMPs may have a detrimental effect on dentinal bonding, contributing to the degradation of the hybrid layer [45, 46].

The loss of pulpal vitality opens the door to a further series of histological and structural alterations that may strongly affect the longevity of an adhesive indirect restoration. The ETT are weakened after root canal treatment and should ideally be covered with indirect partial or full restorations [47]. The amount of remaining tooth structure, the marginal ridge maintenance, the functional occlusal forces, and the quality of the dentin substrate have significant associations with the longevity of the coronal restoration [48–50].

According to a retrospective cohort study, the overall survival rates of ETT without crown coverage at 1, 2, and 5 years are 96%, 88%, and 36%, respectively [47]. Therefore, the placement of an indirect restoration should be strongly recommended for posterior ETT [51]. Nevertheless, the cuspal coverage of vital teeth affected by large II class or MOD cavities with reduced residual dentin thickness could be considered beneficial for long-term prognosis [52].

A full crown restoration remains the most proven solution in the literature, showing high longevity with the biological cost of a more invasive dental preparation [14, 53]. According to modern literature, the sacrifice of a less sound tooth structure in preparing a partial adhesive indirect restoration compared with full crown coverage may be a determinant for the long-term tooth prognosis [14, 53, 54]. Nowadays, the modern adhesive systems allow a minimally invasive approach both for vital and ETT teeth, with the purpose of preserving tooth tissues and protecting dental health, while simultaneously restoring esthetic and function [55].

Therefore, less invasive bonded partial restorations, such as onlays and overlays, have been suggested as valid treatment options for ETT [17, 56]. Recent studies have reported that their longevity depends directly on the amount of remaining tooth structure and the efficacy of restorative procedures in replacing fracture structural integrity [57]. Hence, studies continue to focus more on partial adhesive restoration, which ensure higher sound tissue preservation than classic fixed full crowns that require additional removal of sound tooth tissue [55].

In this scenario, direct resin composite restorations represent the least invasive approach possible. To date, resin composites have reached a deep improvement, with the goal of increasing biomechanics behavior and, in conjunction with effective bonding techniques [58], reducing the need for indirect adhesive restoration. However, resin composites still have some limitations in terms of mechanical properties. The clinical outcomes of direct resin composite restorations vary in the literature, ranging from catastrophic to acceptable [59, 60]. Nevertheless, even when maintaining as much dental tissue as possible, clinical results are seriously influenced by the maintenance of long-term proper adhesion [61]. However, the adhesive systems may have different behaviors on non-vital enamel and dentin substrates [62–64]. Moreover, previous studies demonstrated that the cervical region enamel may negatively influence the bonding quality compared to occlusal or mid-coronal enamel, which appears less vulnerable to micro-leakage [65]. Thus, the longevity of indirect partial adhesive restorations on ETT has been investigated, highlighting the influence of several peculiar factors on long-term outcomes, such as the quantity and quality of the remaining dental tissues [66].

These factors may represent an important difference between vital and non-vital teeth, leading to possible clinical consequences related to the quantity and quality of enamel and dentin substrates [67]. Recently, different technologies and materials have been proposed for the creation of these types of partial adhesive restorations, such as CAD-CAM systems and composite or ceramic materials [68]. Therefore, one of the main objectives of this systematic review was to investigate the survival rate of partial indirect bonded restorations on posterior vital and endodontically treated teeth.

The present study showed that partial adhesive restorations seemed more favorable on vital teeth than on ETT; thus, the initial null hypothesis was rejected.

The limitations of this meta-analysis could be found in the homogeneity of the included studies: they were not randomized prospective clinical trials that directly compared the survival rates of indirect adhesive restorations. The follow-ups did not have the same duration and ranged from a minimum of 9.5 years to a maximum of 16.7. Moreover, the materials with which the indirect restorations were made are not always the same. These differences are all sources of heterogeneity. The inclusion of these studies was based on previous meta-analyses conducted on the topic but with different outcomes. From the qualitative and quantitative analyses, it emerged that the studies comparing the failure rates of inlays, onlays, and overlays on vital and non-vital teeth were only eight.

Moreover, data regarding long-term outcomes were often missing and divergent. The 2010 study by van Dijken et al. reported a prospective study with an average 15-year follow-up for partial ceramic coverages and described 16 failures out of 41 in non-vital teeth compared with 39 out of 186 in vital ones [35]. Reiss et al. (2006) reported the highest odds ratio of inlay failures in 28 out of 77 teeth compared with 94 out of 934 in vital teeth [23]. Apparently, Schulte et al. (2005) reported slightly different data: the odds ratio was 0.66, although in the forest plot (Fig. 4), the data was not statistically significant where the confidence intervals intersected the line of non-effect [38]. Similarly, Beier et al. (2012) [34] and Bresser et al. (2019) [36] reported no statistically significant failure data for the secondary outcome. In particular, Beier et al. (2012) reported 5 failures in 9 onlays and inlays against 23 failures in 538 indirect partial adhesive restorations on vital teeth with 12 years of follow-up, but the non-vital teeth population seemed too small to draw significant conclusions [34].

However, the lack of systematic long-term clinical data in the literature is partially justified by the considerable scientific effort that clinical trials represent. Moreover, the continuous evolution of clinical techniques may be responsible for the relatively difficult organization of the obtained scientific data. Regarding the pooled hazard ratio, the evaluated studies seemed all significant, and the value of 7.78 was partially confirmed by the high value of the pooled odds ratio.

The most frequent reasons for the failure of indirect partial composite restorations have been reported to be secondary caries and fractures of the restorative material and the non-covered cusps [52]. Moreover, the partial indirect ceramic restorations showed the predominant risk of loss of retention [54]. Therefore, the clinical durability of dentin-bonded restorations is strongly dependent on the degradation of the restorative material and the luting agent [46, 59]. The adhesive bonding of restorations to dentin has been indicated to be weak and technique-sensitive [60, 69], especially for the proximal margins where the majority of secondary caries are diagnosed [60]. Typically, a tooth that requires endodontic treatment has lost a large volume of tissue and is more prone to fracture [15], besides having a more reduced retention area compared to vital teeth [54].

The loss of marginal ridges has been shown to reduce cuspal stiffness and, in the case of MOD cavities, this is to an extent of 63% [48]. Loss of water and weakened collagen cross-linking [70] strongly affect frailty through increased cuspal deflection during function, with a consequent higher occurrence of fractures [57, 71]. Even endodontic irrigants have a negative impact on the physical properties of dentin, which makes it less suitable as a bonding substrate [67]. The difference in adhesive substrate to which the primers are applied may be predominant: hydrophilic dentin in vital teeth and sclerotic less water-containing dentin tissues in ETT [35]. Therefore, non-vital teeth are usually less prone to perceive increased load if not properly restored [72]. The adhesive techniques allow the preservation of residual tooth tissue, avoiding the creation of micromechanical retentions, but vital teeth characteristics are usually more favorable for long-term indirect adhesive partial restorations outcomes [73].

## Conclusions

Within the limitations of this study, the risk of failure seems much higher for indirect partial adhesive restorations on ETT than for those on vital teeth.
